# Effect of 3 Different Daily Protein Intakes in a 2-Meal Eating Pattern on Protein Turnover in Middle Age and Older Adults: A Randomized Controlled Trial

**DOI:** 10.1016/j.tjnut.2024.12.025

**Published:** 2024-12-28

**Authors:** David D Church, Katie R Hirsch, Shiloah A Kviatkovsky, Joseph J Matthews, Reino A Henderson, Gohar Azhar, Robert R Wolfe, Arny A Ferrando

**Affiliations:** 1 Department of Geriatrics, Donald W. Reynolds Institute on Aging, Center for Translational Research in Aging and Longevity, University of Arkansas for Medical Sciences, Little Rock, AR, United States; 2 Department of Exercise Science, Arnold School of Public Health, University of South Carolina, Columbia, SC, United States; 3 Department of Orthopaedic Surgery, University of Arkansas for Medical Sciences, Little Rock, AR, United States

**Keywords:** time-restricted eating, protein synthesis, muscle, sarcopenia, lean body mass

## Abstract

**Background:**

Reduced meal frequency patterns have become popular for weight loss, maintenance, and improving cardiometabolic health. The extended fasting windows with these dietary patterns could lead to greater protein breakdown, which is a concern for middle-aged and older adults who may need higher protein intakes to maintain or increase net protein balance.

**Objectives:**

This study aimed to quantify muscle and whole-body protein kinetic responses to 3 different daily protein intakes within a 2-meal eating pattern.

**Methods:**

Thirty participants (age: 61 ± 6 y, BMI: 26.5 ± 4.8 kg/m^2^) participated in this 24-h metabolic study using oral stable isotope tracer techniques and were randomized to 1 of 3 protein intakes: *1)* recommended dietary allowance (RDA): 0.8 g/kg/d; *2)* habitual United States intake: 1.1 g/kg/d; or *3)* ≈2RDA: 1.5 g/kg/d distributed across 2 meals, consumed within a 9-h window.

**Results:**

Whole-body net protein balance was significantly higher for 1.5 g/kg/d compared with 0.8 g/kg/d [mean difference: 0.55 g/kg; lean body mass (LBM)/d; 95% confidence interval (CI): 0.17, 0.93 g/kg LBM/d; *P* = 0.004] and 1.1 g/kg/d (mean difference: 0.6 g/kg LBM/d; 95%CI: 0.23, 0.97 g/kg LBM/d; *P* = 0.001), with no difference between 0.8 and 1.1 g/kg/d (mean difference: 0.05 g/kg LBM/d; 95%CI: −0.31, 0.40 g/kg LBM/d; *P* = 0.936). Muscle protein synthesis was not significantly different between any groups (*P* = 0.388).

**Conclusion:**

*s*: Within a 2-meal eating pattern, a protein intake of 1.5 g/kg/d led to a more positive whole-body net protein balance than intakes of 0.8 and 1.1 g/kg/d in middle-aged and older adults.

This trial was registered at clinicaltrials.gov as NCT04830514.

## Introduction

Reduced meal frequency patterns have become popular for weight loss, maintenance, and improving cardiometabolic health [[1], [2], [3]]. These approaches alter the feeding–fasting cycle with the duration between the first and last energy intake typically restricted to 6–10 h. Older adults are more likely to follow a 2-meal eating pattern, whether purposefully or unintentionally, with data showing ∼25%–48% regularly skip ≥1 meal per day (most commonly lunch) [[4], [5], [6]]. A potential issue with these eating patterns is that the longer fasting window leads to a greater time spent in a catabolic state, increasing protein breakdown. Because protein does not have an inactive component to serve as a reservoir, an increased postabsorptive period could potentially lead to a net negative 24-h protein balance, which over time could lead to losses in lean body mass (LBM) [3]. These concerns are heightened in older adults as they generally have less muscle mass than younger individuals and are more susceptible to sarcopenia, morbidity, and ultimately, mortality [7].

Dietary protein intake stimulates protein turnover and helps to maintain muscle mass, functional capacity, and quality of life in aging adults [8]. The recommended dietary allowance (RDA) for protein is 0.8 g/kg/d, defined as the minimum intake necessary to avoid a negative nitrogen balance [9]. National Health and Nutrition Examination Survey (NHANES) data show that middle-aged and older adults habitually consume 1.0–1.1 g/kg/d [10]. However, consumption of >1.1 g/kg/d may be insufficient in this demographic to maintain protein balance, as the anabolic response to protein decreases with age [11]. To address this and to reduce the risk of chronic disease and age-related muscle loss, some consensus committees recommend protein intakes ≤1.5 g/kg/d [12,13]. Further recommendations are to evenly distribute protein across 3 meals, based on the theory of maximally stimulating muscle protein synthesis (MPS) with each meal (∼0.4–0.5 g/kg/meal) [3,14] Meeting these dietary goals may pose a problem for reduced meal frequency patterns; for example, a 2-meal eating pattern requires ∼0.55 and ∼0.75 g protein/kg/meal to meet intakes of 1.1 and 1.5 g/kg/d and the “excess” dietary protein per meal may not be taken up by skeletal muscle [15,16].

Consistent with the potential difficulty of meeting optimal protein intake with a 2-meal feeding pattern, some controlled feeding studies show that evenly distributed protein intake (e.g., ∼30 g per meal across 3 meals or ∼20 g per meal across 4 meals) leads to greater 24-h MPS than skewed protein intake (∼10, ∼15, and ∼65 g per meal distribution or ∼40 g per meal across 2 meals) [14,17]. In contrast, other studies show that daily protein distribution does not affect the anabolic response, provided that adequate total protein intake is achieved and ≥1 meal maximally stimulates MPS [[18], [19], [20], [21], [22]]. When considering longer fasting windows, consuming 1.0 g/kg/d of protein across 3 meals within an 8-h time-restricted eating window led to similar rates of daily MPS to those following a traditional 12-h eating window [20]. However, it is unclear if the expected improvement in protein balance would occur with a 2-meal pattern and dietary protein intakes that exceed the per meal recommendations. It is possible that higher protein intakes might increase whole-body protein synthesis, suppress whole-body protein breakdown, and promote a more positive whole-body protein net balance than intakes of ∼0.4 g/kg/meal [23]. If protein balance can be improved with dietary protein intakes above current per meal recommendations, then the 2-meal eating pattern may enable adequate dietary protein consumption to meet even the highest recommendations. This study aimed to assess the effect of 3 different daily protein intakes (0.8, 1.1, and 1.5 g/kg/d) in a 2-meal eating pattern (2 meals consumed within a 9-h window) on skeletal muscle and whole-body protein turnover in middle-aged and older adults.

## Methods

### Participants

Participants were required to be 50–70 y old with a BMI ≤ 35 kg/m^2^ and considered generally healthy; all women were considered postmenopausal. Participants were excluded if they *1)* had complete blood count laboratory results that indicated anemia or abnormal white blood cell counts; *2)* had a history of chemotherapy or radiation in the 6 mo prior; *3)* were using insulin to control blood glucose levels; *4)* were currently receiving androgen (e.g., testosterone) or anabolic (e.g., growth hormone) therapy; *5)* were currently using prescription blood-thinning medications; or *6)* were unable or unwilling to consume animal protein sources. All participants provided written informed consent prior to participation.

### Experimental design

**FIGURE 1:**
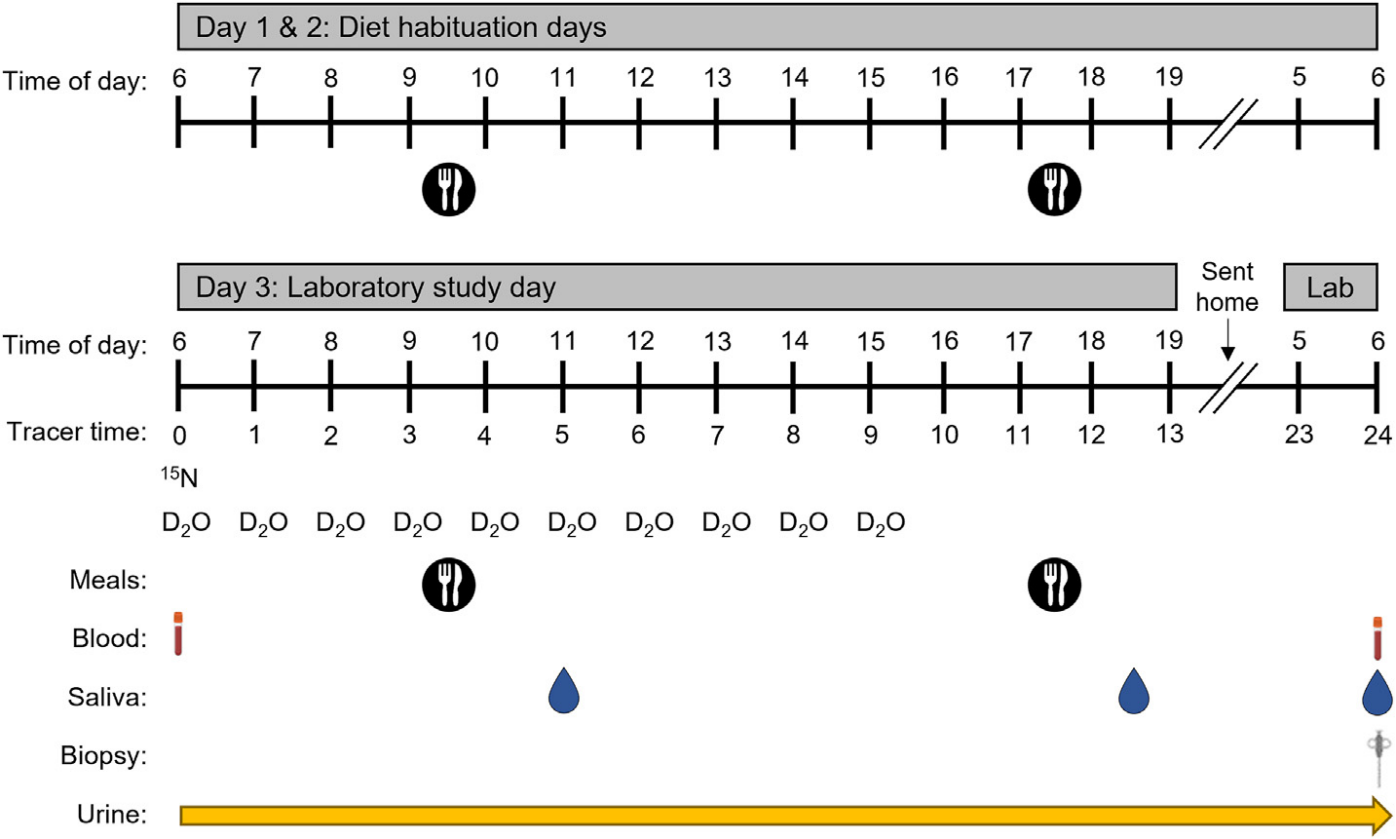
Study experimental design. Participants received a 2-d habituation diet that provided the same energy and macronutrient intake as the metabolic study day. Participants were recommended to follow the same mealtimes on habituation days. D_2_O, deuterated water; ^15^N, ^15^N-alanine.

In a randomized parallel-groups design, a 24-h metabolic study using oral stable isotope tracer techniques was conducted to quantify integrated whole-body and muscle protein turnover responses to 1 of 3 protein intakes: *1)* RDA: 0.8 g/kg/d; *2)* habitual US intake according to NHANES [10]: 1.1 g/kg/d; or *3)* ≈2RDA 1.5 g/kg/d. Dietary intake was standardized for a 2-d run-in period prior to and throughout the 24-h testing period (3-d total) (Figure 1). Participants remained in the laboratory throughout the study day and were allowed home overnight, returning the next morning to complete a final blood draw, saliva sample, and muscle biopsy. Participants were instructed to arrive for each testing visit fasted (10 h overnight), to avoid vigorous physical activity for 24-hours prior to the study day, and to abstain from taking aspirin and protein/amino acid supplements whereas participating in the study. Body composition was determined from a dual-energy X-ray absorptiometry whole-body scan (QDR-4500A; Hologic) and bioelectrical impedance analysis (InBody770, BioSpace). Isotope tracers were purchased from Cambridge Isotope Laboratories. Metabolic studies were completed at the Reynolds Institute on Aging at the University of Arkansas for Medical Sciences (UAMS), and all procedures were approved by the UAMS Institutional Review Board.

#### Meals

Participants were provided with 2 d of ready-to-eat meals (2 meals/d) to consume at home prior to the study visit. Study day meals were all served and consumed between 09:30 and 10:30 (meal 1) and 17:30 and 18:30 (meal 2). Meals were designed to mimic a standard American diet and prepared by a dietitian in the Metabolic Kitchen at UAMS (sample menu provided in Supplemental Table 1). Individual caloric requirements were estimated using the Harris–Benedict equation based on participant age, height, weight, and gender [24]. Protein amount was based on the assigned group, with carbohydrates and fat provided to meet remaining caloric requirements. Participants were instructed to follow similar mealtimes to the study day and eat only the food that was provided. They were allowed to add noncaloric condiments and seasonings to the meals and consume noncaloric beverages; all eaten and uneaten foods were recorded on written and photographic compliance logs. Dietary nitrogen was quantified from both study meals on day 3 to account for nitrogen intake [25] in the determination of whole-body protein turnover.

### Stable isotope tracer protocols

#### Whole-body protein turnover (^15^N-alanine)

Whole-body protein turnover (g N/24-h) was determined by [^15^N]alanine isotope tracer (98% enriched, Cambridge Isotope Lab) in which participants consumed a single 2-g oral dose mixed with water [26]. For the 24-h following ingestion, participants collected each urine void. Zero and 24-h blood draws were collected to measure residual ^15^N-urea in the body pool. Isotopically labeled nitrogen in urea from the blood and urine samples was used to determine nitrogen flux according to Fern et al. [27]. Whole-body protein synthesis and breakdown were calculated from urine samples according to Stein et al. [28] and used to determine net protein balance and flux. The following equations were used to calculate whole-body protein synthesis, breakdown, net balance, and flux:

(1) $$\text{Flux}=\frac{\left(\text{Dose}\;\text{of}\;\left[15N\right]\text{alanine}\right)\times \left(\left(\text{urine}\;\text{urea}\;\text{nitrogen}\times \text{total}\;\text{urine}\;\text{volume}\right)-\left({\text{BUN}}_{24}-{\text{BUN}}_{0}\right)\right)}{\left(\left(\text{Urine}\;\text{urea}\;\text{nitrogen}\times \text{total}\;\text{urine}\;\text{volume}\right)\times E_{\text{Urine}}\right)+\left(\left({\text{BUN}}_{24}\times {\;E}_{\text{Plasma}}\right)\right)}$$

(2) Proteinsynthesis=flux−totalurinarynitrogen(=urinaryureanitrogen×1.1)

(3) Proteinbreakdown=flux−dietarynitrogeniIntake

(4) Netbalance=proteinsynthesis−proteinbreakdown

Where E is enrichment of [^15^N]alanine in the respective compartment expressed as tracer-to-tracee ratio (TTR), and BUN is blood urea nitrogen. Total nitrogen was derived by measuring urinary urea nitrogen and multiplying by 1.1, as 90% of urinary nitrogen is excreted in the form of urea [29]. Whole-body protein kinetic outcomes are described as units per kilogram of LBM.

#### Muscle protein synthesis

A percutaneous muscle biopsy was obtained from the vastus lateralis under local anesthesia (1% lidocaine) as previously described [30]. Muscle protein synthesis was determined by oral D_2_O ingestion to assess integrated changes over a 24-h period using the single biopsy approach, which yields the same values as the more traditional multiple biopsy approaches, provided the incorporation time is >4 h [31]. The D_2_O dose (0.005 mL per kg of total body water × 0.65) was divided into 10 aliquots and consumed every hour for the first 10 h of the study visit to minimize the risk of dizziness and nausea that can occur with D_2_O ingestion [32]. The fractional synthetic rate (FSR) was determined from the incorporation of D_2_O-labeled alanine into muscle protein, using the enrichment of body water, corrected for the mean number of deuterium moieties incorporated per alanine, as the surrogate precursor labeling. MPS (%/d) was calculated as −ln(1 − *f*)/*t*, where (*f*) is *f* is calculated as protein-bound alanine TTR divided by the precursor pool and (*t*) the duration of label [33,34]. Body water was used as the precursor enrichment instead of the free alanine enrichment to have an integrated value over the entire study period.

### Analytic methods

Alanine nitrogen enrichments in blood and urine were measured via GC-MS (models 7890A/5975; Agilent Technologies). Ions of the *m/z* of 231 and 232 for alanine and 238 and 241 for methyl-histidine were monitored with electron impact ionization and selective ion monitoring. Muscle samples were weighed, and tissue proteins precipitated with 0.5 mL of 4% sulfosalicylic acid (SSA), then homogenized, centrifuged, and the muscle pellet (bound protein) was washed, dried, and hydrolyzed in 0.5 mL of 6 N HCl at 105°C for 24 h. Muscle preparations were analyzed for incorporation of deuterated alanine in muscle tissue by liquid chromatography-tandem mass spectrometry (AB SCIEX Triple Quad 4500; AB SCIEX). Body water enrichment following D_2_O ingestion was determined through isotope exchange with acetone using GC-MS [35] (enrichment data are presented in Supplemental Figure 1). Values were recorded in triplicate, and the intra-assay coefficient of variation for D_2_O-bound protein, saliva D_2_O, serum [^15^N]alanine, and urine [^15^N]alanine enrichments were 0.71%, 2.7%, 0.16%, and 0.12%, respectively.

### Statistical analysis

We performed a power calculation using G∗Power software (version 3.1.9.7) for a 1-way analysis of variance (ANOVA) F-test to compare whole-body net protein balance between 3 treatment groups. A sample size of *n* = 10 participants per treatment group was required to detect an effect size (*f*) of 0.60, with a significance level (*α*) of 0.05 and power (1 − *β*) of 0.8. This was based on ≈2RDA whole-body protein balance of 0.6 ± 0.3 g/kg/LBM/d and population mean across protein intakes included in the present study; derived from a large internal database of [^15^N]alanine whole-body protein balance values. Normal distribution was assessed using the Shapiro–Wilk test and visual inspection of Q–Q plots. One-factor ANOVAs were used to compare between-group differences in protein kinetic measures and macronutrient intakes, significant main effects were followed up with Tukey post hoc tests corrected for multiple comparisons. The eta-squared (*η*^2^) statistic was used to describe effect sizes for main effects. Partial Pearson correlation coefficients were used to describe the relationship between protein intake and whole-body net protein balance. Outliers were assessed (1.5 × interquartile range) for whole-body protein balance (*n* = 2) and winsorized to the next less extreme value [36]. All statistical procedures were performed using SPSS (version 26; IBM Corp.). Statistical significance was accepted at *P* < 0.05, and values are presented as mean ± standard deviation or mean difference (MD) with 95% confidence interval (95%CI) unless otherwise stated.

## Results

**FIGURE 2:**
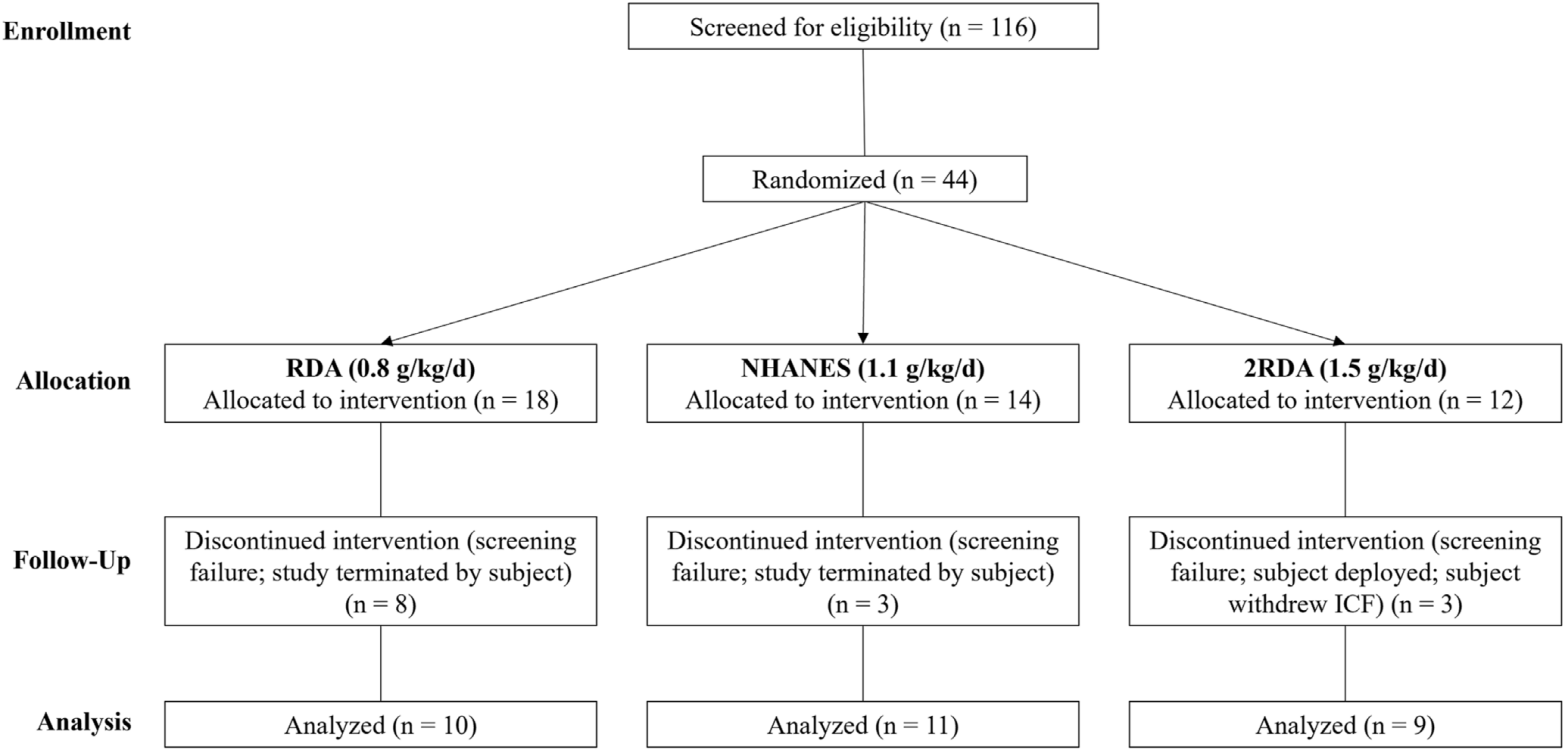
CONSORT flow diagram of study recruitment, enrolment, follow-up, and analysis.

Thirty participants were included in the final analysis (Figure 2). Eight participants were excluded after in-person screening due to screening failures (2 concomitant use of medications, 1 fall risk, 1 abnormal blood count, 2 with several diet sensitivities that could not be accommodated, and 2 with BMI ≥ 35 kg/m^2^); one participant withdrew from the study due to time constraints; 5 participants did not complete the protocol due to nausea associated with D_2_O intake. Complete data were available for *n* = 27 (muscle FSR), and *n* = 28 (whole-body protein kinetics) participants; the exact group *n* is reported in each table and figure legend.

### Demographics and macronutrient intake

There were no significant differences between groups for any demographic data (Table 1). Consistent with the study design, there were differences between groups for protein and carbohydrate intakes (Table 2).

**TABLE 1:** Participant demographics and body composition.

Variable	RDA (*n* = 10)	NHANES (*n* = 11)	2RDA (*n* = 9)
Males/females (*n*)	5/5	5/6	3/6
Age (y)	60.2 ± 7.0	62.5 ± 5.8	60.3 ± 3.6
Mass (kg)	81.4 ± 20.7	72.6 ± 21.0	79.0 ± 18.4
BMI (kg/m^2^)	27.3 ± 5.0	25.0 ± 4.5	27.8 ± 3.9
Fat mass (kg)	25.4 ± 10.2	20.9 ± 8.7	26.4 ± 7.8
Fat-free mass (kg)	53.5 ± 13.2	50.0 ± 14.2	49.5 ± 14.6
Lean body mass (kg)	52.4 ± 12.1	47.9 ± 13.2	47.2 ± 14.0
Percent body fat (%)	31.0 ± 8.1	29.1 ± 7.5	35.2 ± 7.5
Total body water (L)	41.6 ± 9.7	38.1 ± 10.3	38.6 ± 10.3

Values are means ± standard deviation. Dual-energy X-ray absorptiometry data are *n* = 8 in the 2RDA group.

NHANES, National Health and Nutrition Examination Survey (1.1 g/kg/d); RDA, recommended dietary allowance (0.8 g/kg/d); ≈2RDA (1.5 g/kg/d).

**TABLE 2:** Dietary intake on the controlled laboratory study day.

Nutrient	RDA	NHANES	2RDA
Absolute intakes			
Calories (kcal)	1954 ± 431	1810 ± 455	1917 ± 453
Protein (g)	66 ± 16^a^	81 ± 23^a^	124 ± 30^b^
Carbohydrate (g)	253 ± 51^a^	219 ± 49^a,b^	198 ± 46^b^
Fat (g)	80 ± 20	71 ± 20	73 ± 18
Relative intakes			
Energy (kcal/kg)	24.3 ± 1.6	25.2 ± 2.0	23.3 ± 1.4
Protein (g/kg)	0.8 ± 0	1.1 ± 0	1.5 ± 0
Carbohydrate (g/kg)	3.2 ± 0.3	3.1 ± 0.4	2.4 ± 0.2
Fat (g/kg)	1.0 ± 0.1	1.0 ± 0.1	0.9 ± 0.1

Values are means ± standard deviation; Values not sharing a similar letter are significantly different from one another (*P* < 0.05).

NHANES, National Health and Nutrition Examination Survey; RDA, recommended dietary allowance (0.8 g/kg/d); ≈2RDA, 1.5 g/kg/d.

### Whole-body protein turnover

**FIGURE 3:**
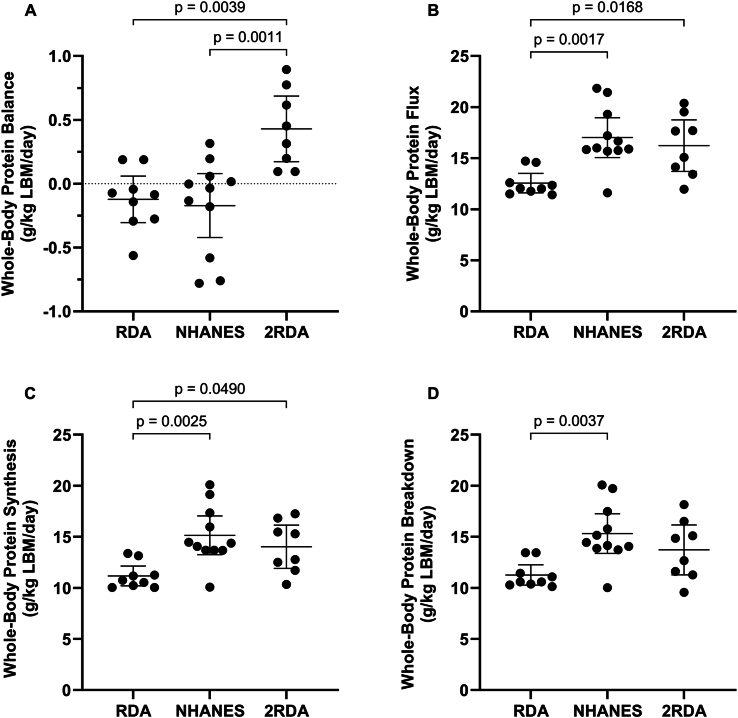
(A) Whole-body net protein balance; (B) whole-body protein flux; (C) whole-body protein synthesis; (D) whole-body protein breakdown. g/kg , grams per kilogram; LBM, lean body mass; NHANES, National Health and Nutrition Examination Survey protein intake (1.1 g/kg/d); RDA, recommended dietary allowance (0.8 g/kg/d); ≈2RDA, 1.5 g/kg/d. Data are presented for *n* = 9 (RDA), *n* = 11 (NHANES), and *n* = 8 (2RDA) participants per group. Values are mean ± 95% confidence intervals.

**FIGURE 4:**
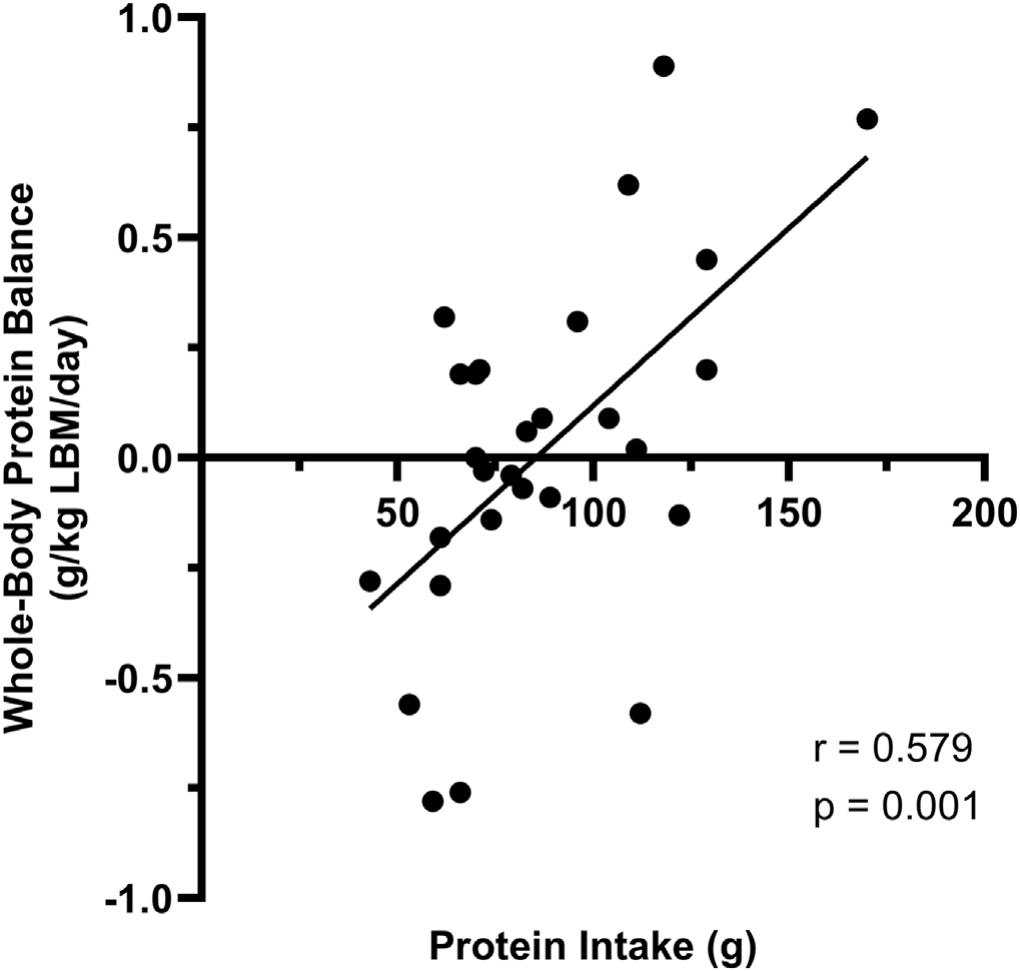
Relationship between protein intake and whole-body net protein balance. g/kg, grams per kilogram; LBM, lean body mass.

There was a significant (*P* < 0.001; *η*^2^ = 0.435) main effect of the group on whole-body net protein balance (Figure 3A). Post hoc tests showed that whole-body net protein balance was significantly higher in ≈2RDA compared with RDA (MD: 0.55 g/kg LBM/d; 95%CI: 0.17, 0.93 g/kg LBM/d; *P* = 0.004) and NHANES (MD: 0.6 g/kg LBM/d; 95%CI: 0.23, 0.97 g/kg LBM/d; *P* = 0.001). There were no differences between RDA and NHANES (MD: 0.05 g/kg LBM/d; 95%CI: −0.31, 0.40 g/kg LBM/d; *P* = 0.936). Whole-body net protein balance was significantly correlated with total protein intake (*P* = 0.001; *r* = 0.579) (Figure 4).

There was a significant (*P* = 0.002; *η*^2^ = 0.400) main effect of the group on whole-body protein flux (Figure 3B). Post hoc tests showed that whole-body protein flux was significantly higher in ≈2RDA compared with RDA (MD: 3.7 g/kg LBM/d; 95%CI: 0.6, 6.7 g/kg LBM/d; *P* = 0.017) and NHANES compared with RDA (MD: 4.5 g/kg LBM/d; 95%CI: 1.6, 7.3 g/kg LBM/d; *P* = 0.002). There were no differences between ≈2RDA and NHANES (MD: −0.8 g/kg LBM/d; 95%CI: −3.7, 2.1 g/kg LBM/d; *P* = 0.782).

There was a significant (*P* = 0.003; *η*^2^ = 0.369) main effect of group on whole-body protein synthesis (Figure 3C). Whole-body protein synthesis was significantly higher in ≈2RDA compared with RDA (MD: 2.9 g/kg LBM/d; 95%CI: 0.0, 5.7 g/kg LBM/d; *P* = 0.049) and NHANES compared with RDA (MD: 4.0 g/kg LBM/d; 95%CI: 1.3, 6.6 g/kg LBM/d; *P* = 0.003). There were no differences between ≈2RDA and NHANES (1.1 g/kg LBM/d; 95%CI: −1.6, 3.8 g/kg LBM/d; *P* = 0.568).

There was a significant (*P* = 0.005; *η*^2^ = 0.343) main effect of the group on whole-body protein breakdown (Figure 3D). Post hoc tests showed that whole-body protein breakdown was significantly higher in NHANES compared with RDA (MD: 4.1 g/kg LBM/d; 95%CI: 1.3, 6.9 g/kg LBM/d; *P* = 0.004). There were no differences between ≈2RDA and RDA (MD: 2.5 g/kg LBM/d; 95%CI: −0.6, 5.5 g/kg LBM/d; *P* = 0.128) or ≈2RDA and NHANES (MD: −1.6 g/kg LBM/d; 95%CI: −4.5, 1.3 g/kg LBM/d; *P* = 0.369).

### Muscle protein synthesis

**FIGURE 5:**
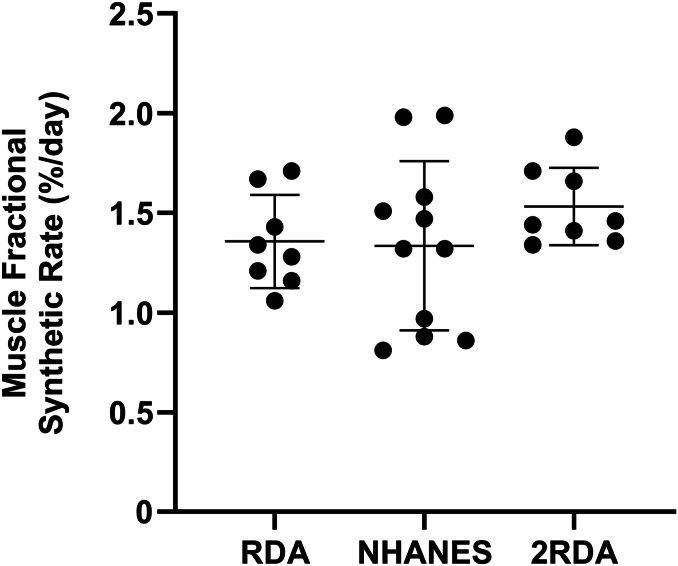
Muscle fractional synthetic rate over the 24-h study period. NHANES, National Health and Nutrition Examination Survey protein intake (1.1 g/kg/d); RDA, recommended dietary allowance (0.8 g/kg/d); ≈2RDA, 1.5 g/kg/d. Data are *n* = 8 (RDA and 2RDA) and *n* = 11 participants per group (NHANES). Values are mean ± 95% confidence intervals.

There was no significant (*P* = 0.388; *η*^2^ = 0.076) main effect of group on muscle FSR (Figure 5); ≈2RDA compared with RDA (MD: 0.18 %/d; 95%CI: −0.22, 0.57 %/d; *P* = 0.525), ≈2RDA compared with NHANES (MD: 0.20 %/d; 95%CI: −0.17, 0.57 %/d; *P* = 0.393), NHANES compared with RDA (MD: −0.02 %/d; 95%CI: −0.39, 0.35 %/d; *P* = 0.988).

## Discussion

The main finding from the present study is that within a 2-meal eating pattern, positive whole-body net protein balance was only achieved with 1.5 g protein/kg/d, approximately double the RDA in middle-aged and older adults. The positive protein balance was ∼17% of the total protein intake, which is consistent with previous studies quantifying nitrogen balance when protein intake exceeds the RDA [37]. Protein balance was significantly higher with 1.5 g protein/kg/d compared with intakes of 0.8 g/kg/d (current RDA) and 1.1 g/kg/d (habitual US intake), despite the higher intake exceeding the typical per meal recommendations for protein. Total protein intake was positively correlated with whole-body net protein balance, which collectively suggests that protein intakes above current recommendations are required to increase protein turnover in middle-aged and older adults consuming a 2-meal eating pattern.

Several studies show that whole-body net protein balance increases linearly with dietary protein intake, even when intake exceeds the RDA [9,22,[38], [39], [40]]. Results from whole-day dietary patterns have, however, been restricted to traditional eating patterns, involving 3 or more meals and/or snacks [22,40]. Here we show that within a 2-meal eating pattern, RDA protein intake did not lead to positive whole-body net protein balance, despite participants consuming ∼0.4 g/kg/meal, which is reported to maximally stimulate MPS [41]. Increasing protein intake from 0.8 to 1.1 g/kg/d (∼0.55 g/kg/meal) did not increase protein balance; this is concerning given that it represents the habitual daily protein intake of middle-aged and older adults in the United States [10]. Furthermore, the whole-body protein balance in these 2 groups is contrary to our previous tracer infusion studies which showed a positive net protein balance [18,22]. The apparent differences in these studies could be explained by the measuring periods. Previous studies of the response to 3 meals per day were performed over 12.5 h, whereas the present study was over a 24-h period. Whole-body protein balance displays a diurnal pattern, whereby overnight postabsorptive losses are balanced by postprandial gains to maintain net balance over the entire day. Thus, if an additional 11.5-h nonfeeding period was included in the measurement period of Kim et al. [22] it is likely that individuals provided the RDA would have experienced a neutral or negative whole-body protein balance. Furthermore, the 2-meal eating pattern not only reduces the number of meals eaten but also shortens the eating window to offset overnight losses and may explain the whole-body protein balance in both the 0.8 and 1.1 g/kg/d groups in our study.

However, our data are consistent with the seminal work of Rand et al. [9], which was the basis for setting the RDA for protein. Their data relating N-balance to dietary N intake showed that most individuals were in negative N-balance when protein intake was <∼1.26 g/kg/d. This agrees with recent work that suggests that a protein intake of 1.2 g/kg/d is required for an N-balance of 0 in trained young men [40]. In the present study, most participants in the 0.8 and 1.1 g/kg/d groups experienced negative whole-body protein balance. Despite this, group effects showed that an overall negative protein balance did not occur with these intakes, potentially due to the small sample size. Because middle-aged and older adults are less responsive to dietary protein than younger individuals, our data showing that higher protein intakes are required for positive whole-body net protein balance is not surprising. Finally, none of the studies on protein intake and feeding patterns have considered the potential impact of protein digestibility and essential amino acid profiles, which could affect the relationship between dietary protein intake and nitrogen or whole-body protein balance.

In contrast to whole-body protein balance, there were no significant differences between groups in MPS. Although this disagrees with previous findings from our group and others [22,42], the between-group mean differences for ≈2RDA and RDA (0.18 %/d) and ≈2RDA and NHANES (0.20 %/day) were similar to previous studies. Deuterium-derived measures of MPS are increased by 0.14%/day in young women when protein intake is increased from 0.80 to 1.59 g/kg/d [42]. Similarly, we previously found L-[ring-2H5] phenylalanine-derived estimates to be 0.23%/d higher in individuals consuming 1.5 g/kg/d compared with individuals consuming 0.8 g/kg/d [15]. It is therefore possible that between-group differences in the present study would have become statistically significant with a larger sample size. In addition, unlike our previous tracer infusion studies, participants in this study were not under laboratory-controlled conditions for the entire 24-h measurement period, and this may have increased variability.

This study has several strengths, including the use of a multitracer approach in the setting of a strictly controlled diet with a 2-d habituation period. Changes in protein kinetics were quantified over a 24-h period, which provides insight into overall protein balance and moves beyond single-meal postprandial measurements. Furthermore, the sample included a split of males and females, which enables translation to both genders. Regarding limitations, it is possible that the study was underpowered to detect changes in skeletal MPS, partly due to increased variability with the 24-h measurement period. Lastly, our results are specific to the 2-meal dietary pattern and protein intakes included, and it is unclear if these effects would occur with longer feeding windows.

In conclusion, consuming 1.5 g/kg/d (∼0.75 g/kg/meal) led to significantly greater whole-body net protein balance compared with intakes of 0.8 and 1.1 g/kg/d in middle-aged and older adults. The greater net protein balance with the consumption of 1.5 g/kg/d was achieved despite exceeding the amount of dietary protein (∼0.4 g/kg/meal) reported to maximally stimulate MPS in a single meal. The practical aspect of this conclusion is that within a 2-meal eating pattern, a large amount of dietary protein is required per meal to achieve a positive whole-body net protein balance over 24 h.

## Author contributions

The authors’ responsibilities were as follows – DDC, KRH, AAF, RRW: conception of the idea, planning of the experiments, and interpretation of results; DDC, KRH, RAH, GA: assisted with data collection; DDC, JJM: led the data analysis; DDC, KRH, JJM: original drafting of the manuscript; and all authors: have read and approved the final manuscript.

## Data availability

The data sets generated and analyzed in the current study are available from the corresponding author upon reasonable request.

## Funding

This study was funded by a grant from the Foundation for Meat and Poultry Research and Education, a contractor to the Beef Checkoff to AAF. DDC is currently supported by a National Institutes of Health (NIH) Clinical Research Loan Repayment Award. Research reported in this publication was supported by the National Center for Advancing Translational Sciences of the National Institutes of Health under Award Number TL1 TR003109 and UL1 TR003107. The content is solely the responsibility of the authors and does not necessarily represent the official views of the National Institutes of Health.

## Conflict of interest

The authors report no conflicts of interest.

## References

[1] Sutton E.F., Beyl R., Early K.S., Cefalu W.T., Ravussin E., Peterson C.M. (2018). Early time-restricted feeding improves insulin sensitivity, blood pressure, and oxidative stress even without weight loss in men with prediabetes. Cell Metab..

[2] Stratton M.T., Albracht-Schulte K., Harty P.S., Siedler M.R., Rodriguez C., Tinsley G.M. (2022). Physiological responses to acute fasting: implications for intermittent fasting programs. Nutr. Rev..

[3] Lee M.J., Hodson N., Moore D.R. (2021). A muscle-centric view of time-restricted feeding for older adults. Curr. Opin. Clin. Nutr. Metab. Care..

[4] Stalling I., Albrecht B.M., Foettinger L., Recke C., Bammann K. (2022). Meal patterns of older adults: results from the OUTDOOR ACTIVE study. Nutrients.

[5] Krok-Schoen J.L., Archdeacon Price A., Luo M., Kelly O.J., Taylor C.A. (2019). Low dietary protein intakes and associated dietary patterns and functional limitations in an aging population: a NHANES analysis. J. Nutr. Health Aging.

[6] Kant A.K. (2018). Eating patterns of US adults: meals, snacks, and time of eating. Physiol. Behav..

[7] Mayhew A.J., Phillips S.M., Sohel N., Thabane L., McNicholas P.D., de Souza R.J. (2020). The impact of different diagnostic criteria on the association of sarcopenia with injurious falls in the CLSA. J. Cachexia Sarcopenia Muscle.

[8] Wolfe R.R. (2006). The underappreciated role of muscle in health and disease. Am. J. Clin. Nutr..

[9] Rand W.M., Pellett P.L., Young V.R. (2003). Meta-analysis of nitrogen balance studies for estimating protein requirements in healthy adults. Am. J. Clin. Nutr..

[10] Berryman C.E., Lieberman H.R., Fulgoni V.L., Pasiakos S.M. (2018). Protein intake trends and conformity with the Dietary Reference Intakes in the United States: analysis of the National Health and Nutrition Examination Survey, 2001–2014. Am. J. Clin. Nutr..

[11] Moore D.R., Churchward-Venne T.A., Witard O., Breen L., Burd N.A., Tipton K.D. (2015). Protein ingestion to stimulate myofibrillar protein synthesis requires greater relative protein intakes in healthy older versus younger men, J. Gerontol. A Biol. Sci. Med. Sci..

[12] Bauer J., Biolo G., Cederholm T., Cesari M., Cruz-Jentoft A.J., Morley J.E. (2013). Evidence-based recommendations for optimal dietary protein intake in older people: a position paper from the PROT-AGE Study Group. J. Am. Med. Dir. Assoc..

[13] Deutz N.E.P., Bauer J.M., Barazzoni R., Biolo G., Boirie Y., Bosy-Westphal A. (2014). Protein intake and exercise for optimal muscle function with aging: recommendations from the ESPEN expert group. Clin. Nutr..

[14] Mamerow M.M., Mettler J.A., English K.L., Casperson S.L., Arentson-Lantz E., Sheffield-Moore M. (2014). Dietary protein distribution positively influences 24-h muscle protein synthesis in healthy adults. J. Nutr..

[15] Højfeldt G., Bülow J., Agergaard J., Asmar A., Schjerling P., Simonsen L. (2020). Impact of habituated dietary protein intake on fasting and postprandial whole-body protein turnover and splanchnic amino acid metabolism in elderly men: a randomized, controlled, crossover trial. Am. J. Clin. Nutr..

[16] Højfeldt G., Bülow J., Agergaard J., Simonsen L.R., Bülow J., Schjerling P. (2021). Postprandial muscle protein synthesis rate is unaffected by 20-day habituation to a high protein intake: a randomized controlled, crossover trial. Eur. J. Nutr..

[17] Areta J.L., Burke L.M., Ross M.L., Camera D.M., West D.W.D., Broad E.M. (2013). Timing and distribution of protein ingestion during prolonged recovery from resistance exercise alters myofibrillar protein synthesis. J. Physiol..

[18] Kim I.-Y., Schutzler S., Schrader A.M., Spencer H.J., Azhar G., Wolfe R.R., Ferrando A.A. (2018). Protein intake distribution pattern does not affect anabolic response, lean body mass, muscle strength or function over 8 weeks in older adults: a randomized-controlled trial. Clin. Nutr..

[19] Agergaard J., Justesen T.E.H., Jespersen S.E., Tagmose Thomsen T., Holm L., van Hall G. (2023). Even or skewed dietary protein distribution is reflected in the whole-body protein net-balance in healthy older adults: a randomized controlled trial. Clin. Nutr..

[20] Justesen T.E.H., Jespersen S.E., Tagmose Thomsen T., Holm L., van Hall G., Agergaard J. (2022). Comparing even with skewed dietary protein distribution shows no difference in muscle protein synthesis or amino acid utilization in healthy older individuals: a randomized controlled trial. Nutrients.

[21] Parr E.B., Kouw I.W.K., Wheeler M.J., Radford B.E., Hall R.C., Senden J.M. (2023). Eight-hour time-restricted eating does not lower daily myofibrillar protein synthesis rates: a randomized control trial. Obesity (Silver Spring).

[22] Kim I.-Y., Schutzler S., Schrader A., Spencer H., Kortebein P., Deutz N.E.P. (2015). Quantity of dietary protein intake, but not pattern of intake, affects net protein balance primarily through differences in protein synthesis in older adults. Am. J. Physiol. Endocrinol. Metab..

[23] Kim I.-Y., Deutz N.E.P., Wolfe R.R. (2018). Update on maximal anabolic response to dietary protein. Clin. Nutr..

[24] Harris J.A., Benedict F.G. (1918). A biometric study of human basal metabolism. Proc. Natl. Acad. Sci. U. S. A..

[25] Wolfe R.R., Rutherfurd S.M., Kim I.-Y., Moughan P.J. (2016). Protein quality as determined by the digestible indispensable amino acid score: evaluation of factors underlying the calculation. Nutr. Rev..

[26] Ferrando A.A., Lane H.W., Stuart C.A., Davis-Street J., Wolfe R.R. (1996). Prolonged bed rest decreases skeletal muscle and whole body protein synthesis. Am. J. Physiol..

[27] Fern E.B., Garlick P.J., Waterlow J.C. (1985). Apparent compartmentation of body nitrogen in one human subject: its consequences in measuring the rate of whole-body protein synthesis with 15N. Clin. Sci (Lond)..

[28] Stein T.P., Rumpler W.V., Leskiw M.J., Schluter M.D., Staples R., Bodwell C.E. (1991). Effect of reduced dietary intake on energy expenditure, protein turnover, and glucose cycling in man. Metabolism.

[29] Weiner I.D., Mitch W.E., Sands J.M. (2015). Urea and ammonia metabolism and the control of renal nitrogen excretion. Clin. J. Am. Soc. Nephrol..

[30] Church D.D., Pasiakos S.M., Wolfe R.R., Ferrando A.A. (2019). Acute testosterone administration does not affect muscle anabolism. Nutr. Metab (Lond)..

[31] Burd N.A., West D.W., Rerecich T., Prior T., Baker S.K., Phillips S.M. (2011). Validation of a single biopsy approach and bolus protein feeding to determine myofibrillar protein synthesis in stable isotope tracer studies in humans. Nutr. Metab. (Lond)..

[32] Jones P.J., Leatherdale S.T. (1991). Stable isotopes in clinical research: safety reaffirmed. Clin. Sci. (Lond)..

[33] Evans W., Shankaran M., Nyangau E., Field T., Mohammed H., Wolfe R. (2021). Effects of Fortetropin on the rate of muscle protein synthesis in older men and women: a randomized, double-blinded, placebo-controlled study. J. Gerontol. A Biol. Sci. Med. Sci..

[34] Gasier H.G., Fluckey J.D., Previs S.F. (2010). The application of 2H_2_O to measure skeletal muscle protein synthesis, Nutr. Metab (Lond)..

[35] Yang D., Diraison F., Beylot M., Brunengraber D.Z., Samols M.A., Andersonet V.E. (1998). Assay of low deuterium enrichment of water by isotopic exchange with [U-13C3]acetone and gas chromatography-mass spectrometry. Anal. Biochem..

[36] Moore D.R., Williamson E.P., Hodson N., Estafanos S., Mazzulla M., Kumbhare D. (2022). Walking or body weight squat “activity snacks” increase dietary amino acid utilization for myofibrillar protein synthesis during prolonged sitting. J. Appl. Physiol. Am. Physiol. Soc..

[37] Hegsted D.M. (1978). Assessment of nitrogen requirements. Am. J. Clin. Nutr..

[38] Church D.D., Hirsch K.R., Park S., Kim I.-Y., Gwin J.A., Pasiakos S.M. (2020). Essential amino acids and protein synthesis: insights into maximizing the muscle and whole-body response to feeding. Nutrients.

[39] Gorissen S.H.M., Trommelen J., Kouw I.W.K., Holwerda A.M., Pennings B. (2020). Protein type, protein dose, and age modulate dietary protein digestion and phenylalanine absorption kinetics and plasma phenylalanine availability in humans. J. Nutr..

[40] Gaine P.C., Pikosky M.A., Martin W.F., Bolster D.R., Maresh C.M., Rodriguez N.R. (2006). Level of dietary protein impacts whole body protein turnover in trained males at rest. Metabolism.

[41] Moore D.R., Robinson M.J., Fry J.L., Tang J.E., Glover E.I., Wilkinson S.B. (2009). Ingested protein dose response of muscle and albumin protein synthesis after resistance exercise in young men. Am. J. Clin. Nutr..

[42] Oikawa S.Y., Bahniwal R., Holloway T.M., Lim C., McLeod J.C., McGlory C. (2020). Potato protein stimulates muscle protein synthesis at rest and with resistance exercise. Nutrients.

