# Supplementary material for: Effect of 3 Different Daily Protein Intakes in a 2-Meal Eating Pattern on Protein Turnover in Middle Age and Older Adults: A Randomized Controlled Trial
Source: J Nutr. 2024 Dec 28;155(5):1364–72. doi: 10.1016/j.tjnut.2024.12.025 (PMC12121411; doi:10.1016/j.tjnut.2024.12.025)
Supplement: Multimedia component 1 [file mmc1.docx]

| **Supplementary Table 1.** Example NHANES group study day meal composition. Portion sizes were changed accordingly to individualize energy and macronutrient intakes within and between groups. | | | | |
| --- | --- | --- | --- | --- |
| **Food item** | **Energy (kcal)** | **Protein (g)** | **Fat (g)** | **Carbohydrate (g)** |
| *Example NHANES group lunch (meal one)* |  |  |  |  |
| Beef, patty ground steak burger, cooked | 153 | 5.2 | 2.4 | 27.2 |
| Rolls, hamburger, plain | 147 | 10.7 | 11.2 | 0.9 |
| Cheese, provolone | 3 | 0.2 | 0.2 | 0.3 |
| Mustard, prepared, yellow | 86 | 0.1 | 9.5 | 0.4 |
| Salad dressing, mayonnaise, soybean oil, with salt | 219 | 2.6 | 15.0 | 19.9 |
| Snacks, potato chips, plain, salted | 88 | 3.0 | 0.8 | 20.7 |
| Corn, sweet, yellow, frozen, kernels cut off cob | 218 | 2.0 | 8.9 | 32.7 |
| Cookies, vanilla wafers, higher fat | 119 | 7.8 | 4.7 | 11.1 |
| Milk, reduced fat, fluid, 2% milkfat, with added vitamin A | 239 | 19.9 | 18.3 | 0.0 |
| **Total meal energy and macronutrient intake** | **1272** | **51.6** | **71.0** | **113.2** |
|  | | | | |
| *Example NHANES group dinner (meal two)* |  |  |  |  |
| Chicken breast, boneless, cooked sous vide, grilled | 223.5 | 41.2 | 5.3 | 1.2 |
| Potato, chunk, herb garlic roasted | 180.0 | 3.6 | 5.4 | 28.8 |
| Carrots, frozen, unprepared | 43.2 | 0.9 | 0.6 | 9.5 |
| Peas, green, frozen, unprepared | 73.2 | 5.0 | 0.4 | 12.9 |
| Rolls, dinner, plain, commercially prepared | 155.0 | 5.4 | 3.2 | 26.0 |
| Cake, pound, commercially prepared, butter | 232.8 | 3.3 | 11.9 | 29.3 |
| Peaches, canned, juice pack, solids and liquids | 77.0 | 1.1 | 0.1 | 20.3 |
| Apple juice, canned or bottled, unsweetened | 138.0 | 0.3 | 0.4 | 33.9 |
| **Total meal energy and macronutrient intake** | **1123** | **60.8** | **27.2** | **161.9** |
| **Total daily energy and macronutrient intake** | **2395** | **112.5** | **98.2** | **275** |


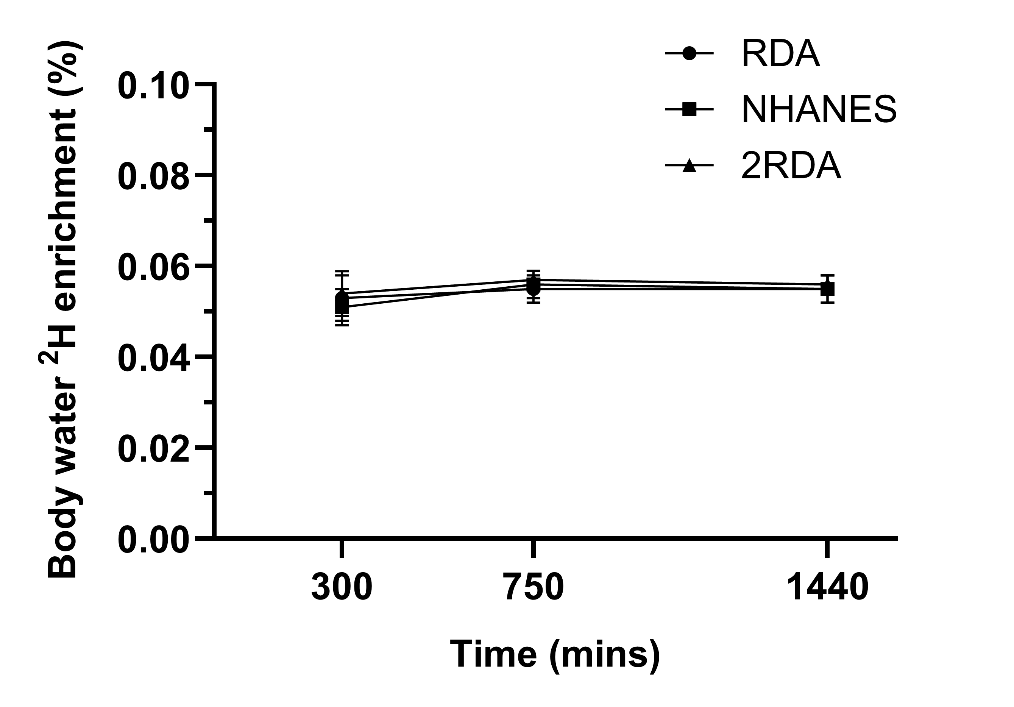


**Supplementary Figure 1.** Body water ^2^H enrichment for each group (*n*=8 per group for RDA and 2RDA; *n*=9 for NHANES). Data presented as mean ± SD.
